# Toxic Effects of Arsenic on Four Freshwater Aquatic Species and Its Transformation Metabolism in Crucian Carp (*Carassius auratus*)

**DOI:** 10.3390/toxics12030221

**Published:** 2024-03-17

**Authors:** Shizhan Tang, Lei Gao, Dongli Qin, Haitao Wang, Li Huang, Song Wu, Shuyan Bai, Ningning Du, Yanchun Sun, Peng Wang, Zhongxiang Chen

**Affiliations:** 1 Heilongjiang River Fisheries Research Institute, Chinese Academy of Fishery Sciences, Harbin 150070, China; tangshizhan@hrfri.ac.cn (S.T.); gaolei@hrfri.ac.cn (L.G.); qindongli@hrfri.ac.cn (D.Q.); wanghaitao@hrfri.ac.cn (H.W.); huangli@hrfri.ac.cn (L.H.); wusong@hrfri.ac.cn (S.W.); baishuyan@hrfri.ac.cn (S.B.); duningning@hrfri.ac.cn (N.D.); sunyanchen@hrfri.ac.cn (Y.S.); 2 Supervision, Inspection and Testing Center for Fishery Environment and Aquatic Products (Harbin), Ministry of Agriculture and Rural Affairs, Harbin 150070, China

**Keywords:** arsenic, freshwater aquatic species, toxicity, arsenic form, exposed, biotransformation

## Abstract

Inorganic arsenic is a well-known carcinogen that is much more toxic than its organic counterpart. While much is known about the accumulation and transformation of arsenic in marine organisms, little is known regarding these processes in freshwater aquatic species. In this study, the acute toxicity and toxicological effects of inorganic arsenic on four freshwater organisms (*Cyprinus carpio*, *Misgurnus anguillicaudatus*, *Pseudorasbora parva*, *Eriocheir sinensis*) commonly found in rice-fish farming systems were investigated. The organisms exhibited different levels of sensitivity to inorganic arsenic, with crustaceans being more sensitive than fish. Fish were found to be more tolerant to As(V) than As(III). The study also investigated the accumulation, transformation, and release of inorganic arsenic in crucian carp, an omnivorous species with high environmental tolerance. The fish accumulated As(III) rapidly in various tissues, and were able to transport it to other tissues through gills, intestines, and skin. The accumulated As(III) was converted into less toxic forms, such as monomethylarsonic acid (MMA) and dimethylarsinic acid (DMA), via methylation. The fish also converted As(III) into arsenate (AsV) via enzymatic and oxidative reactions. After the transferal to clean water, the forms of arsenic in the various tissues decreased rapidly, but the rates of excretion of the four forms of arsenic were not the same among the different tissues. Our results suggest that crucian carp can reduce the environmental toxicity of As(III) at certain concentrations by transforming it into less toxic forms within their bodies.

## 1. Introduction

Arsenic (As) is widely distributed in nature, and is absorbed through the food chain and bioaccumulation, leading to the formation of various arsenic species in organisms through methylation. Among all arsenic species, inorganic arsenic (iAs), including arsenite (AsIII) and arsenate (AsV), is the most toxic form, and long-term exposure to low levels of iAs can cause cancer and other health hazards to in humans [1,2]. The genetic toxicity of iAs metabolites, such as monomethylarsonic acid (MMA) and dimethylarsinic acid (DMA), is much lower. However, DMA has been identified as a promoter of iAs-induced carcinogenesis. Other organic arsenic compounds, such as arsenobetaine (AsB) and arsenocholine (AsC), are considered non-toxic [3,4].

In recent years, As has been widely spread in aquatic environments due to both natural and anthropogenic factors. It not only causes toxicity to aquatic organisms, but also transfers via the food chain to higher trophic levels, including humans. This has become a serious global environmental issue in more than 20 countries, and the groundwater of over 75 countries is contaminated with As, exposing over 160 million people (especially those in rural areas) [5]. In China, the scale of the integrated rice–fish farming industry has steadily increased, with the farming area exceeding 39 million acres and a yield of over 3.5 million tons in 2021 [6]. However, there are widespread toxic As substances, such as arsenite, in the rice field environment [7]. Issues such as high detection rates of As in aquatic products and dietary risks have gradually attracted widespread attention [8]. The species in the rice-fish co-culture mode mainly include common carp (*Cyprinus carpio* L.), crucian carp (*Carassius auratus*), loach (*Misgurnus anguillicaudatus*), red swamp crayfish (*Procambarus clarkia*), and Chinese mitten crab (*Eriocheir sinensis*), while the topmouth gudgeon (*Pseudorasbora parva*) is often used in the form of bait. Existing studies have found that cadmium (Cd) and As are the heavy metal species that pose the highest risks of harm, and iAs poses the greatest threat. Different freshwater fish species exhibit different levels of tolerance to iAs [9]. Shellfish have a higher risk of acute and chronic stress due to their specific physiological characteristics and benthic living habits, with a lower avoidance rate of common pollutants. Crucian carp, as a freshwater benthic species, are often used as a model for biotransformation and toxicity research [10], while loach has a shorter lifecycle and a faster response to environmental pollutants, making it a typical test species for evaluating the toxicological effects of pollutants [11]. In aquatic toxicology, traditional LC50 tests are often used to measure the potential risks of chemicals. By searching the US Environmental Protection Agency’s ECOTOX database, we found that there are few studies on the acute toxicity of iAs to loach, topmouth gudgeon, Chinese mitten crab, and crucian carp, and only a few species have been tested. The LC50 values of 96 h of As(III) for *Labeo rohita*, *Cirrhina mrigala*, *Catla catla*, and *Ctenopharyngodon idella* were 30.0 ± 0.0, 24.5 ± 0.1, 10.2 ± 0.2, and 22.2 ± 0.0 mg/L, respectively [12]. The impact of iAs on specific aquatic biological toxicological systems remains to be clarified, and further research on biological accumulation, transformation, and metabolism patterns also lacks.

In freshwater and marine fish, As mainly exists in less toxic organic forms. iAs in freshwater fish usually accounts for 1% to 20% of the total As, but under As pollution conditions, the proportion of iAs in freshwater fish can be higher than 20%, which indicates that the existent form of As in water may affect the development of the As form in fish tissues [13]. However, current research is mainly focused on the biotic accumulation and tissue distribution of marine fish, and there is relatively little research on the transformation process of the As form in freshwater fish tissues [14,15]. Therefore, studying the dynamic changes of As biotic accumulation and transformation in freshwater fish during chronic As exposure is particularly important, highlighting the important reference significance for further research on the toxicity and bioaccumulation of As.

The study aimed to assess the acute toxicity of iAs exposure on four freshwater aquatic species and explore the impacts of chronic exposure on bioaccumulation, biotransformation, and antioxidant responses, hypothesizing that the antioxidant stress response from As species interconversion during chronic exposure could be a key factor in As toxicity in freshwater fish. Comparative analysis was conducted between the3 acute toxicity test results and reference values from the US Environmental Protection Agency’s ECOTOX database to evaluate species sensitivity. Subsequently, a 30-day exposure experiment with 1 mg/L As(III) in crucian carp was carried out using a semi-static bioassay, followed by an investigation into the changes in As bioaccumulation kinetics, speciation, and antioxidant enzyme responses following the exposure and subsequent release of fish into clean water for 10 days. The study is expected to serve as a foundational reference for iAs toxicity research in crucian carp, contributing new insights into the ecotoxicological effects of highly toxic compounds on freshwater aquatic organisms.

## 2. Materials and Methods

### 2.1. Experimental Materials

Preparation of the test fish, *C. auratus* (48.0 ± 2.5 g), *M. anguillicaudatus* (11.4 ± 1.7 g), *P. parva* (2.4 ± 0.3 g), and *E. sinensis* (78.7 ± 5.3 g), were purchased from a local aquatic products wholesale market in Harbin city. Healthy individuals with uniform specifications were selected as experimental subjects, and were kept in aquariums for one week prior to the experiment, with a mortality rate below 1%. During the acclimation period, the fish were fed daily with the feed amount being equivalent to 2% of their body weight. Half of the water was replaced with aerated diluted water (aerated for more than 3 days). Feeding was stopped 24 h before the experiment.

The preparation of iAs solution was as follows: a solution of As(III) was prepared by dissolving sodium arsenate (NaAsO_2_, Sigma, Allentown, PA, USA), and a solution of As(V) was prepared by dissolving sodium arsenate (Na_2_HAsO_4_·7H_2_O, Sigma, Allentown, PA, USA) for acute toxicity testing.

### 2.2. Acute Toxicity Test

In all experiments, for use as the test water, tap water was aerated for 24 h with a heating device to maintain the temperature at 23 ± 1 °C. A commercial air pump (S-4000, Boyu, Chaozhou, China) was used to aerate the water in order to maintain the dissolved oxygen (DO) levels close to saturation (>7.0 mg/L). The pH value was maintained at 7.4 ± 0.2, as measured using a desktop pH meter (pHS-3C, Rayleigh, Shanghai, China). Other water quality parameters were also measured as follows: conductivity (DDS-320, Kangyi, Shanghai, China), between 0.389 and 0.407 mS/cm; German hardness of 250 mg/L (as CaCO_3_); ammonia between 0.22 and 0.25 mg/L; nitrite between 0.003 and 0.005 mg/L; and nitrate between 0.12 and 0.15 mg/L. To confirm that they met the nominal values, water samples were collected from each test tank at the beginning and end of the acute toxicity test, and iAs concentrations were determined using high-performance liquid chromatography coupled with inductively coupled plasma mass spectrometry (HPLC-ICP-MS) (Table S3).

The test containers for the loaches and crucian carp were organic glass fish tanks with a volume of 20 L (40 × 20 × 25 cm), with 15 L of test solution added to each tank. The test containers for crucian carp and Chinese mitten crabs were organic glass fish tanks with a volume of 60 L (50 × 30 × 40 cm), with 50 L of test solution added to each tank. Prior to the experiment, the test chemicals were prepared as a stock solution using standard dilution water and added to the fish tanks in the required proportions. A preliminary test was conducted to determine the highest possible concentration of iAs (As(III) 0.1 mg/L, As(V) 1 mg/L) that did not cause any deaths and the lowest possible concentration (As(III) 200 mg/L, As(V) 1000 mg/L) that caused 100% mortality in all four freshwater species. Based on this concentration range, six test concentration gradients and one blank control were set up with equal logarithmic intervals, with three replicates at each concentration level and 20 fish per replicate.

Following the initiation of the test, the activity status, symptoms, and time of death of the fish were continuously observed and recorded for 8 h. The number of deaths was counted at 24, 48, and 96 h for each group, and any dead individuals were removed promptly. Death was determined based on non-responsiveness to a needle prick and the cessation of gill movements. The light–dark cycle of the semi-static bioassay method was set at 16:8. In order to minimize the impact of the test species on mucus shedding, the test solution was changed every 12 h.

### 2.3. Arsenic Toxicology Experiment

Crucian carp (length 13–15 cm, weight 60–80 g) were obtained from a fish farm in Heilongjiang Province, China. They were kept in aerated tap water (15 °C), and fed 2% of their body weight daily. One week before the exposure experiment, feeding was stopped, and the fish were temporarily housed. The As(III) solution was prepared using sodium arsenite (NaAsO_2_ Sigma, Allentown, PA, USA) with an As(III) concentration of 1 mg/L (based on acute toxicity test results, which showed that concentrations below 3.18 mg/L were safe for crucian carp). Eighty crucian carp were placed in organic glass fish tanks with a volume of 640 L (60 × 60 × 180 cm), each containing 400 L of test solution. This procedure was replicated three times, with a control group being set up concurrently. The fish were exposed to the solution for 30 days with a light–dark ratio of 1:1, and the solution was changed every 24 h. They were then released into clean water for 10 days. During the experiment, the fish were fed for one hour per day, and any fish excrement was promptly removed from the tank. Water samples were collected weekly both before and after the water changes to monitor the stability of the exposure concentration. The actual concentration in the control group was not detectable (LOD 0.27 μg/L), while that in the As(III) exposure group was 0.98 ± 0.15 mg/L. No fish deaths were observed during the exposure period.

Three fish were collected from both the experimental and control groups on days 1, 3, 5, 7, 10, 15, and 30 of the exposure period, and on days 1, 3, 5, 7, and 10 of the release period to measure the concentrations of As(III), As(V), MMA, and DMA. The fish were washed with pure water before sampling and then placed in sealed polyethylene bags. The standard length, total length, and wet weight of each fish were measured immediately. Samples of skin, gills, intestines, liver, and muscle were collected and then frozen at −80 °C for further analysis. 

After the experiment, the remaining individuals were anesthetized with ether and then sacrificed. All experimental animals and used solutions were collected and handed over to a waste disposal company for unified treatment.

### 2.4. Arsenic Speciation Analysis

Samples of fish tissues including gills, skin, intestines, liver, and muscles were thawed on ice, homogenized, and then stored in small polyethylene bags at −20 °C for total As and As speciation analysis.

Total As analysis was carried out as follows: 0.2 g of each sample was accurately weighed on an analytical balance and digested with 2.5 mL of 65% nitric acid, 0.5 mL of 37% hydrochloric acid, and 7.0 mL of ultrapure water. Microwave digestion steps were followed Tang et al. (2023) [16]. After digestion, the solution was transferred to a 50 mL volumetric flask, and 0.5 mL of internal standard solution (100 μg/L) was added to a fixed volume of 50 mL. A blank solution was also prepared. The total As content of the samples was analyzed via inductively coupled plasma mass spectrometry (ICP-MS, Agilent 7500C, Table S1). The accuracy of the digestion method was validated by analyzing the certified reference material BCR627 tuna tissue, and the total As recovery rate was 97.1% (Table S2).

As speciation analysis was performed using high-performance liquid chromatography-inductively coupled plasma mass spectrometry (HPLC-ICP-MS), following the procedures described by Nawrocka et al. (2022) [17]. The chromatographic column (Hamilton PRP-X100 4.1 mm × 250 mm, 10 μm) was purchased from Beijing Haiguang Instrument Co., Ltd., Beijing, China. The limits of detection (LOD) for As(III), As(V), DMA, and MMA were obtained through the analysis of the blank solution in seven replicates, following the method used by the US Environmental Protection Agency (2011) [18]. The LOD were 4.5 μg/kg for As(III), 5.0 μg/kg for As(V), 3.2 μg/kg for DMA, and 1.5 μg/kg for MMA. Method blanks were analyzed for each batch of samples. As spike recovery rates were 95.5–103.6% for As(III), 85.2–96.8% for As(V), and 93.1–105.8% for MMA. The recovery rate for DMA analysis using BCR-627 tuna tissue was 72.5–86.3% (Table S2).

### 2.5. Biokinetic Model of Arsenic Bioaccumulation

A two-compartment toxicokinetic model was employed to simulate the uptake and elimination of As(III) in fish [19]. The model assumed that As introduced into the first compartment of water could be reversibly transferred to the second compartment, excluding direct excretion. The two phases of the experiment were described as follows:

Accumulation phase: (0 < t < t*),

(1) $$C_{A}=C_{0}+C_{W}\frac{k_{1}}{k_{2}}\left(1-e^{k_{2}t}\right)$$

Metabolism phase: (t > t*),

(2) $$C_{A}=C_{W}\frac{k_{1}}{k_{2}}\left[e^{{-k}_{2}(t-t^{*})}-e^{{-k}_{2}t}\right]$$

where t* denotes the time (30 days) at which the accumulation phase ends, C_0_ represents the As(III) concentration (μg/kg wet weight) in the fish prior to the experiment, C_W_ represents the As(III) concentration (μg/L) in the water, C_A_ represents the As(III) concentration (μg/kg wet weight) in the fish, k_1_ is the rate constant of biological uptake, and k_2_ is the rate constant of biological elimination.

### 2.6. Determination of Oxidative Stress Levels

Five fish were collected from both the experimental and control groups for liver enzymatic activity and malondialdehyde (MDA) content measurements on days 1, 4, 7, 14, and 28 of the exposure period. Briefly, the livers were homogenized on ice to ensure sample homogeneity. All commercial assay kits used in this study were purchased from the Nanjing Jiancheng Bioengineering Institute in China. Malondialdehyde (MDA) was measured using the thiobarbituric acid (TBA) and MDA reaction, which was detected using spectrophotometry at 532 nm. Superoxide dismutase (SOD) activity was measured by inhibiting the yellowing rate of the xanthine/xanthine oxidase system in the presence of superoxide anion radicals generated by the oxidation reaction, detected using spectrophotometry at 550 nm. Acetylcholinesterase (AChE) was determined via the hydrolysis of acetylcholine to generate choline and acetic acid. Choline reacts with a chromogenic reagent to produce a yellow Sym-Trinitrobenzene (TNB) product, which is quantified using a spectrophotometric method at a wavelength of 412 nm. Glutathione S-transferase (GST) activity was measured at 412 nm based on the GST-catalyzed reaction between glutathione (GSH) and the substrate 1-chloro-2,4-dinitrobenzene (CDNB) using a glutathione reductase (GR) cycle. The total protein content in individual fish was determined by the reaction between bicinchoninic acid (BCA) and reduced Cu^+^ using spectrophotometry at 562 nm. Enzyme assays were repeated at least twice. According to the testing method, the sample volume for each assay ranged from 30–100 µL. The homogenate was centrifuged at 3000 rpm for 10 min at 4 °C, and the supernatant was stored at 4 °C. All enzyme assays and MDA content measurements were performed together at the end of the experiment and completed within 12 h.

### 2.7. Statistical Analysis

To calculate the half-lethal concentration (LC50) of iAs in fish and the standard error (Sx50), we referred to the modified Karber method [20,21]. Specifically, we used the following equations:

(3) $$\log{LC}_{50}=X_{m}-\left(\sum p-0.5\right)\;\mathrm{and}\;S_{x50}=i\sqrt{\sum \frac{pq}{n}}$$

where X_m_ represents the logarithm of the maximum dose of the dead group, i represents the logarithmic difference in the concentration between adjacent groups, p represents the mortality rate of each group, ∑p represents the total mortality rate of all groups, q represents the survival rate of each group, and n represents the number of individuals in each group.

The formula for calculating the safe concentration (SC) is as follows [21]:

(4) $$SC=\frac{{48h\;LC}_{50}\times 0.3}{{\left(\frac{{24h\;LC}_{50}}{{48h\;LC}_{50}}\right)}^{2}}$$

The 95% confidence limit (CV) of LC_50_ was calculated using the following formula [20]:

(5) $$C_{v}=\log{LC}_{50}\pm 1.96\times S_{x50}$$

In this study, a nominal concentration of 1 mg/L of As(III) was selected to determine its concentration in different tissues of crucian carp. Using non-linear fitting methods with Origin software, toxicokinetic parameters and their standard deviations were estimated. The absorption rate constant (k_1_), elimination rate constant (k_2_), as well as other biokinetic parameters, including bioconcentration factor (BCF), maximum accumulation concentration (C_Amax_), and half-saturation concentration (B_1/2_), were calculated [22].

(6) $$BCF=\frac{k_{1}}{k_{2}}$$

(7) $$C_{Amax}=BCF\times C_{W}$$

(8) $$B_{1/2}=\frac{In2}{k_{2}}$$

Statistical analysis was performed using SPSS 19.0. One-way analysis of variance (ANOVA) was used to test the differences in the corresponding values between different treatments, followed by least significant difference (LSD) testing. A probability level (*p*-value) less than 0.05 was considered statistically significant. Nonlinear fitting was performed using Origin to estimate the toxicokinetic parameters.

## 3. Results and Discussion

### 3.1. Acute Toxicity Effects

Within the first 8 h of the acute toxicity test, the fish in the low-concentration groups of iAs showed similar behavior to the control group, mostly lying quietly at the bottom of the tank and swimming slowly. In contrast, the high-concentration group showed more intense reactions, with frequent movements, but no abnormal respiration was observed. With the passage of time, the eel’s swimming became slow, the breathing accelerated, and they appeared unbalanced and suspended vertically. There were occasional instances of jumping out of the water, with wide mouth openings and vigorous gill breathing. The As(III) high-concentration group (50–160 mg/L) showed redness in the gills, fins, and genital pores, and the body stiffened at death. The loach mainly showed a lighter body color, white fin rays, accelerated respiration, unbalanced body movement, and bleeding under the fins. At death, the gill cover opened, the gill filaments turned white, the head and dorsal fins were congested, the body stiffened, and the fish floated on the water surface. The behavior of the Chinese mitten crab mainly consisted of staying near the bottom of the tank, breathing quickly, and suffering from limb loss. At death, the reproductive lid was loose. The crucian carp mainly showed accelerated respiration, mucous shedding, unbalanced body movement, and tissue damage. At death, the gill cover opened, the gill filaments turned white, and the fish floated on the water surface.

**Figure 1:**
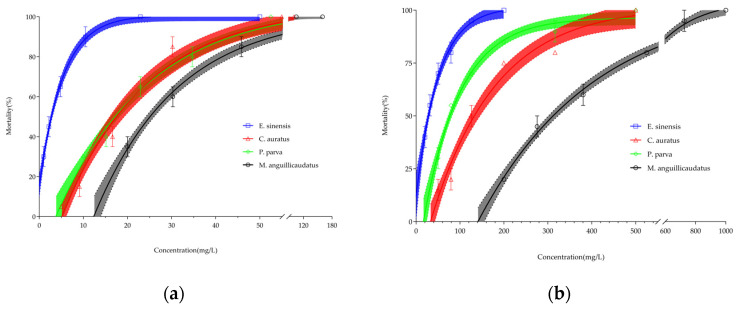
Mortality of *M. anguillicaudatus*, *P. parva*, *E. sinensis*, and *C. auratus* exposed to (**a**) As(III) and (**b**) As(V) for 96 h.

The mortality rates of the four freshwater aquatic species in acute toxicity tests of iAs are shown in Figure 1. Overall, with an increase in test concentration and time, the acute toxicity of iAs on the four freshwater aquatic species significantly increased, and the mortality rate showed a clear upward trend. The acute toxicity response and behavior of the four freshwater aquatic species to As(III)and As(V) mainly manifested as accelerated respiration, unbalanced body movement, changes in body color, and tissue damage.

### 3.2. Inorganic Arsenic’s Half Lethal Concentration and Safe Concentration for Four Freshwater Aquatic Species

The relationship between acute toxicity exposure death rate and logarithmic concentration was analyzed using linear regression equation, as shown in Table 1. It is worth noting that no deaths were observed in any of the replicates of the negative control group. All results were statistically significant (*p* < 0.05, n = 20). The results showed a linear regression relationship between the logarithmic concentration of iAs and the probability unit of the death rate (*p* < 0.05). The regression equations for the four freshwater aquatic species (24 h, 48 h, and 96 h) all showed good positive correlation (the correlation coefficient R was greater than 0.912).

Table 2 presents the safe concentration (SC) of iAs and the estimated LC50 values for each of the four species, along with their respective 95% confidence limits. The results show that, in terms of the toxicity of As(III), the four species ranked in the following order: *E. sinensis* > *C. auratus* ≈ *P. parva* > *M. anguillicaudatus*. For As(V), the toxicity ranking was *E. sinensis* > *P. parva* > *C. auratus* > *M. anguillicaudatus*.

This study also conducted a one-way ANOVA to analyze the LC50 values, which showed that both As(III) and As(V) had a significant impact on the mortality rates of the four freshwater aquatic species (*p* = 0.001), with As(III) exhibiting higher toxicity than As(V). The Tukey HSD test revealed that there was no significant difference in the LC50 values between *P. parva* and *C. auratus* when exposed to As(III) (*p* = 0.997), but there was a significant difference between *E. sinensis* and the other three fish species (*p* = 0.001). There was a significant difference in the toxicity of As(V) among the four species (*p* = 0.014), with *M. anguillicaudatus* showing a significantly higher LC50 value when compared to the other three fish species (*p* < 0.001).

**Figure 2:**
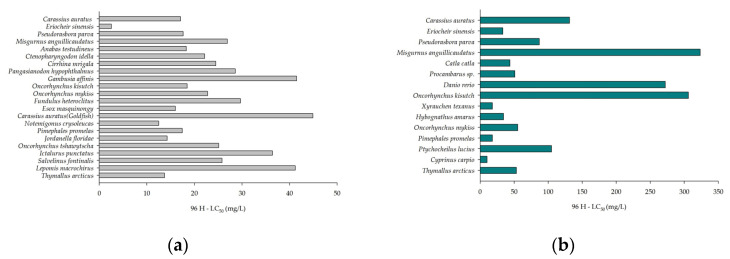
LC50 of arsenic in freshwater organisms. (**a**) represents the LC50 of As(III), and (**b**) represents the LC50 of As(V).

In general, As toxicity levels vary among species. The LC50 of iAs in freshwater aquatic organisms is shown in Figure 2, with data sourced from the U.S. EPA ECOTOX database and literature [23,24]. From Figure 2a, it can be observed that for As(III), the LC50 values of *C. auratus* and *P. parva* in this study are similar to those of *Anabas testudineus*, *Oncorhynchus kisutch*, and *Pimephales promelas*. The LC50 value of *M. anguillicaudatus* is comparable to *Pangasianodon hypophthalmus*, *Fundulus heteroclitus*, *Oncorhynchus tshawytscha*, and *Salvelinus fontinalis*. Among the freshwater fish species retrieved, goldfish (*C. auratus*) exhibit the highest LC50 value, indicating the highest tolerance to As(III). In comparison to freshwater fish, *E. sinensis* shows a higher sensitivity to As(III) with the lowest LC50 value. In Figure 2b, for As(V), the LC50 value of *M. anguillicaudatus* is the highest, demonstrating the highest tolerance to As(V). The LC50 value of *E. sinensis* is similar to *Hybognathus amarus*. Among the freshwater fish species retrieved, *Cyprinus carpio* has the smallest LC50 value, indicating the highest sensitivity to As(V). For *P. parva* and *P. promelas*, their sensitivities are similar, which may be related to them both being scaly omnivorous fish with similar body sizes. Compared to *P. parva* and *P. promelas*, goldfish of the Cyprinidae family have relatively larger individuals and slightly higher sensitivities. Thus, the toxicity of As in organisms is species specific, and may be also affected by many factors like feeding habits and individual size.

Typically, aquatic animals mainly absorb and accumulate As through epidermal penetration, gill respiration, and intestinal absorption. Studies have shown that chitosan in crab shells contains positively charged amino groups, which can easily capture negatively charged As(V) in water, making crabs more prone to As accumulation in their bodies than fish and shrimp [25]. When As concentration in water is low, crabs are often the dominant species for As accumulation [26]; however, when the As concentration in water is high, crabs can rapidly accumulate large amounts of As, leading to significant acute As toxic effects. Previous studies have also shown that As is the most harmful heavy metal in crabs [8,16,27]. 

### 3.3. Biokinetic Parameters of Inorganic Arsenic Bioaccumulation

In general, the interaction process between water and organisms can be described using a two-phase partitioning model. However, the results of this study showed that the ability of crucian carp to accumulate As(III) in various tissues followed the order of liver > skin > intestine > gill > muscle (Table 3). Nonlinear regression fitting was significantly achieved in the muscle, intestine, skin, and liver, with correlation coefficients ranging from 0.87 to 0.89, indicating that the model is suitable. Gills were influenced by both individual and environmental factors, with a low correlation coefficient of 0.45, making these unsuitable for describing the accumulation and metabolism patterns. Additionally, the absorption rate constant values of different tissues ranged from 0.01 to 0.41, while the elimination rate constant ranged from 0 to 0.726.

Similar to our study, Ferreira et al. [28] found that the accumulation order of iAs in different tissues of *Oreochromis niloticus* following a 7-day exposure period was liver > stomach > gill > muscle. The values of the absorption rate constant (k1) in the biota accumulation experiment ranged from 0.06 to 0.51, while the values of the elimination rate constant (k^2^) ranged from 0.03 to 1.15. The lower bioconcentration factor (BCF) values in muscles compared to gills and intestines reflect the distribution pattern of iAs species in the tissues of freshwater fish, which is consistent with our research findings.

The enrichment rate and elimination rate of gills were both the highest, while the enrichment rate of muscle was the lowest, and the elimination rate of liver was null. The experimental results showed that, following exposure to As(III), the crucian carp rapidly accumulated As via the gills, and then rapidly enriched it in various tissues through the blood and other tissues. The null elimination rate of the liver may be due to its function as a detoxification and storage organ, as well as its high accumulation capacity, which results in the highest As(III) content in this tissue. Fish can adapt to chronic As exposure. Chen et al. [29] studied the bioaccumulation and biotransformation of iAs in *Oryzias mekongensis* during chronic exposure and, according to the toxicokinetic data, the low bioaccumulation of As is caused by low absorption rates, internal transfer, and high elimination rates. These findings are consistent with the conclusions of this study. During long-term exposure, the biotransformation capacity of As increases in order to adapt to iAs stress. The rapid conversion and elimination of fish tissues (muscle, gills, and intestine) during As exposure relate to the absorption and elimination rates, which eventually led to a decrease in the bioaccumulation of highly toxic As(III).

### 3.4. Biological Transformation and Metabolism of Arsenic Species in Crucian Carp

**Figure 3:**
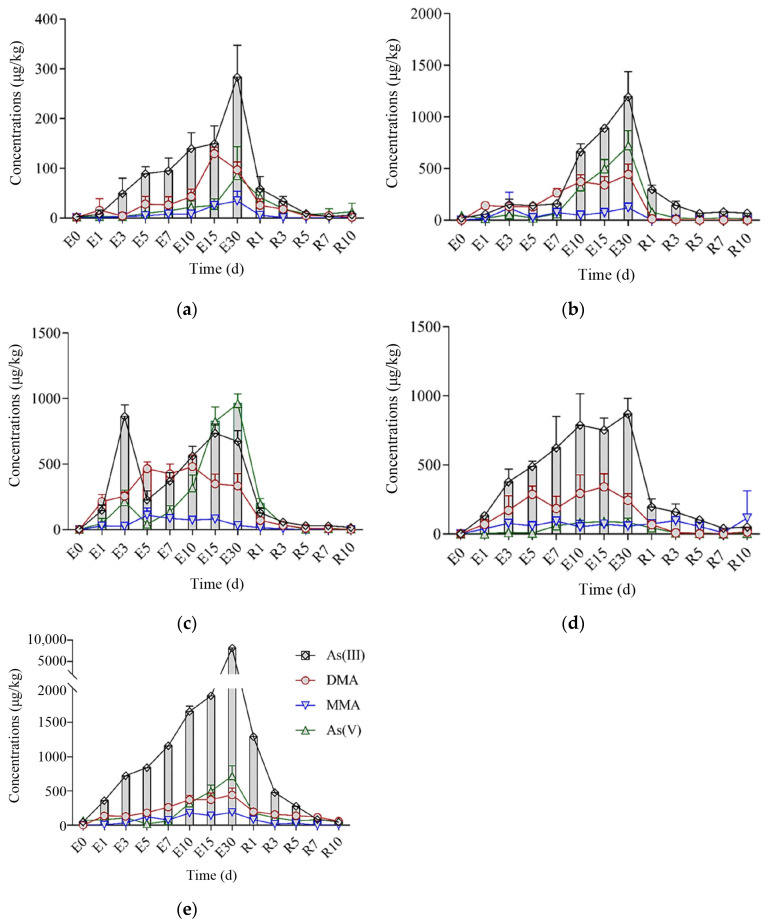
Changes of different forms of arsenic in crucian carp tissues under 1.0 mg/L As(III) stress (n = 3), (**a**) muscle, (**b**) skin, (**c**) gill, (**d**) intestine, (**e**) liver. “E” represents the exposure stage, “R” represents the release stage.

Figure 3a displays the biological enrichment and transformation of As in the muscle tissue of crucian carp following exposure to As(III). Compared with the control group, the concentration of As(III) in the muscle tissue of crucian carp continued to increase after 30 days of exposure to As(III). On the 15th day of exposure, the concentration of DMA in the muscle tissue was significantly higher than on the 10th day (*p* = 0.001) and the 30th day (*p* = 0.037), while the concentration of MMA increased slightly after the 10th day. The increase in As(V) appeared at the end of the exposure period, and the concentration was relatively low. Zhang et al. [30] found that the concentration and proportion of As(III) in the muscle tissue of marine fish increased significantly following exposure to As(V) for 21 days, and As(V) was reduced to As(III). It can be observed that the results of As(V) exposure contrast those of As(III) exposure. Previous studies have confirmed that there is a significant correlation between As(III) and As(V) in fish muscle tissue, and that these can be transformed into each other [3]. However, the specific transformation mechanism is relatively complex, and there is currently no authoritative explanation. From the results of this study, we can speculate that when fish are subjected to As(III) stress in the environment, the body tends to convert As(III) to As(V) [31].

After transferring to clean water, the concentration of DMA decreased to a low level within 3 days, while MMA could decrease to the control group concentration level within 24 h, but As(III) and As(V) were still detected after 10 days. The excretion rate of As(V) in the muscle was relatively slow compared with other forms of As. Once crucian carp were removed from As(III) stress, the concentration of As(III) in the muscle tissue decreased rapidly, and DMA, MMA, and As(V) also decreased. Suhendrayatna et al. [32] found that up to 70%–90% of As was purified by tilapia within 1 day, which further confirms the effect of higher excretion rates on biological accumulation.

Figure 3b displays the bioaccumulation and transformation of As within the skin tissue of crucian carp following exposure to As(III). Compared to the control group, the accumulation of As in fish exposed to As(III) was significantly higher (*p* < 0.01). As(III) accumulation was positively correlated with exposure time, and the increase in As(III) in the skin of crucian carp continued until the 30th day of exposure. DMA was produced during the initial stage of exposure (1–5 days), and remained consistent in concentration, while its concentration fluctuated during subsequent exposure periods. MMA showed higher levels only on the 3rd and 30th day of exposure, and the As(V) concentration increased after the 10th day. It can be speculated that, following As(III) exposure, the skin can continuously accumulate As(III), which is then methylated to DMA, as reported in previous studies [28,30]. The lack of significant changes in MMA levels during the exposure period suggests that the demethylation process of DMA is not strong. Some studies have shown that MMA and DMA are unstable in the body, and can not only be excreted through urine, but can also be converted to AsB [30]. However, it has been demonstrated that freshwater fish cannot produce AsB [33], and the mechanism and content of AsB production in freshwater fish remain unclear. Based on the increase in As(V) concentration after the 10th day, it can be speculated that the oxidation reaction of As(III) is relatively slow and complex. Following the transferal to clean water, DMA, MMA, and As(V) returned to control group levels within 24 h, while As(III) could not be completely metabolized out of the body within ten days. Research has shown that As(V) can be directly excreted from the body, and its metabolism rate is faster than that of As(III), which needs to be gradually eliminated via cellular channels. Additionally, As(V) can be converted to As(III) through a reduction reaction.

Although the toxicity of As(III) is usually higher than that of As(V), various methods can be activated to address the toxicity of As(III) in organisms, including the excretion of As from cells, the conversion to organic As, and the isolation of As(III) within cells through metal-binding peptides, such as metallothionein-like proteins (MTLPs), phytochelatins (PCs), and GSH [19,23,34]. It is reassuring that the concentration of As(III) can be reduced to below the national standard limit within three days of release.

Figure 3c shows the biological accumulation and transformation of As in the gills of crucian carp following exposure to As(III). Toxicity studies on freshwater fish have shown that As is mainly absorbed through the gills in the water phase [35]. Following exposure to As(III), the amount of As biologically accumulated in the gills of crucian carp increased significantly (*P* < 0.01). Among them, the As(III) accumulation in the gills of crucian carp reached its peak on the third day; it increased linearly from days 5 to 15, and remained stable with a slight decrease occurring between days 15 and 30. DMA was produced in the early stages of exposure (1–5 days), and its content was relatively consistent. Subsequently, its content was relatively stable. MMA only had a high content on the third day of exposure and then continued to decrease. MMA is an intermediate in the biological conversion of As(V) to DMA. It can be speculated that the gill tissues of crucian carp undergo methylation in the early stages of As(III) exposure. MMA, as a metabolic intermediate, can be converted to DMA in a short time, resulting in low As(V) content in the early stage and increased MMA and DMA content. This is consistent with the research conclusion of Pei et al. [36] in the As(III) foodborne exposure experiment on tilapia. As(V) linearly increased after the fifth day. Following As(III) stress, the gills can accumulate As(III) in the short term, and As(V) continues to increase after the fifth day, indicating that the gills of crucian carp can convert part of the accumulated As(III) into As(V). The As(III) content decreased from days 15 to 30, while As(V) continued to increase. In the later stage of As(III) stress, the gills can convert As(III) into As(V) via complex physiological mechanisms, while the content of MMA and DMA did not significantly increase (*p* > 0.05).

After the transfer to clean water, the concentration of MMA decreased to the control group level within 24 h, while DMA, As(III), and As(V) could not be completely metabolized out of the body within 10 days. As(III) metabolism was relatively slow, and the conversion efficiency of As(V) and DMA decreased after the As(III) stress was alleviated.

Figure 3d shows the bioaccumulation and transformation of As in the intestine of crucian carp following As(III) exposure. Compared with the control group, the content of As(III) in the crucian carp intestine linearly increased within 10 days of exposure, slightly decreased at the 15 day point, and slightly increased after 30 days. Within 10–30 days of exposure, the content of As(III) in the intestine tended to stabilize, indicating that the absorption and excretion of the intestine tended to stabilize following 10 days of As(III) stress, with almost the same absorption and excretion rates. While the content of As(III) in the intestine increased, the content of DMA continued to increase for the first five days, and fluctuated between days 7–30, while the content of MMA and As(V) did not change significantly. It can be inferred that methylation occurs in the intestine of crucian carp as As(III) accumulates, with an increase being observed in the DMA content. Similar research results have also shown that iAs undergoes oxidation, reduction, and methylation in the intestine of tilapia, with a lower methylation rate than in the liver [36].

Methylation mainly occurred in the early stages of stress, with a slight decrease in As(III) content and a peak in DMA content after 15 days, followed by an increase in As(III) content. It is speculated that methylation in the intestine of crucian carp mainly occurs within 15 days before exposure, possibly due to the fact that the intestine has lower methylation ability than the liver and muscle tissues. In addition, the intestine is the first step in biological transformation, while the liver and muscles absorb it in the form of organic As (with a higher proportion of organic As). Therefore, although there are differences in methylation, the conversion of As(III) from an inorganic form to an organic form (MMA and DMA) in the intestine may be an important process. Throughout the stress period, we observed an increase in the As(V) concentration, indicating that As(III) can also be oxidized to As(V) in the intestine of crucian carp. Such results are rarely reported in fish; this may be due to the high absorption rates of As(V) in the intestine [14]. 

After being transferred to clean water, the As(III) content in the intestine rapidly decreased on the first day, followed by a linear decrease over the next seven days. The elimination rate of As(III) from the intestine was fast once the As(III) stress environment had been left, which suggests that As(III) mainly exists in a water-soluble state [37]. DMA levels decreased to control group levels within 3 days, while MMA levels increased after 10 days, reaching the maximum level during the entire experimental period. The intestine contains a large number of microbial populations. As a result of microbial decomposition activity, As(III) can be converted into MMA and stored in the intestine, leading to an increase in MMA content after 10 days of release [38].

From Figure 3e, it can be observed that during As(III) exposure, there are varying degrees of growth in the content of the four arsenic species in the liver of crucian carp, with As(III) showing the highest increase. The levels of DMA and As(V) are comparable to those in the skin, but significantly higher than those in the muscle and intestines (*p* < 0.05). Upon entering the crucian carp body, As compounds accumulate and undergo mutual transformation in multiple tissues, followed by transport and distribution via the blood. The liver is rich in various detoxifying enzymes, but its excretion rate is relatively low, ultimately leading to a continuous increase in As(III) content in the liver [39]. After being transferred to clean water, the As(III) content in the liver showed a significant downward trend, but its content was still nearly 30 times higher than that of the control group. The excretion rate of As(V) was relatively high, but the content of As(V) in the liver was higher than in other tissues. It is speculated that crucian carp converts a portion of As(III) to As(V) via the action of detoxifying enzymes in the liver [40]. On the other hand, it may also be due to the high affinity between As(III) and thiol, which can bind with the glutathione (GSH) and cysteine present in the liver or blood to generate compounds that can then be excreted through urine.

Our study confirmed the patterns of As accumulation and transformation in different forms the tissues of crucian carp that had been exposed to As(III). The accumulation of As(III) in different tissues varied in response to waterborne exposure. At the beginning of the exposure, As(III) accumulated heavily in the gills and intestines, while the skin tissue showed a significant increase after one week. In the later stages of exposure, the As species underwent mutual transformation, with As(III) in the muscle and intestines mainly converting to DMA, while in the skin and gills, it mainly transformed to As(V). In the late stage of exposure, the rate of As(III) accumulation slowed down in all tissues except the liver, and even decreased in the gills. Meanwhile, the concentrations of As(V), DMA, and MMA fluctuated and gradually reached a steady state. Upon being removed from the stress environment, the As(III) concentration in various tissues rapidly decreased due to the higher elimination rate. The reduction in As(III) concentration in the liver may be related to the biological transformation of As species. Relevant studies have also found that the levels of various As species in both freshwater and marine fish are significantly influenced by adaptive strategies. The differences in As content levels in fish may be associated with the intake of different types of iAs and exposure durations [19,29,30]. When aquatic organisms are exposed to As, they can accumulate high concentrations of As within a short period. Subsequently, through a conversion process, toxic iAs is transformed into less toxic organic As in order to cope with As exposure in the environment [41,42].

### 3.5. Enzymatic Responses and Lipoperoxidation Level

**Figure 4:**
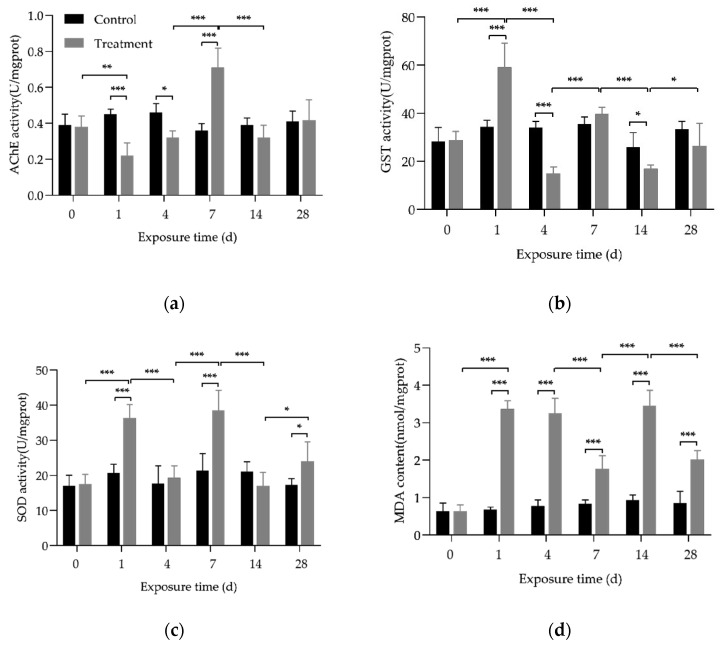
The contents of enzymatic activity in *C. auratus* chronically exposed to 1 mg/L waterborne inorganic As(III) for 28 d. (**a**) AchE, (**b**) GST, (**c**) SOD, and (**d**) MDA. * represents *p* < 0.05, ** represents *p* < 0.01, and *** represents *p* < 0.001.

As shown in Figure 4, on the first day of As(III) exposure, the activities of GST and SOD enzymes, as well as the content of MDA in the liver of crucian carp were significantly higher than those in the control group. (Figure 4b–d, *p* < 0.001), indicating that the fish started to undergo a stress response to As(III) exposure. On the fourth day, SOD was inhibited, GST was lower than the control group (Figure 4b, *p* < 0.001), and MDA remained significantly higher than then normal levels (Figure 4d, *p* < 0.001), indicating that oxidative damage to cell membranes occurred in the early stages of As(III) exposure. On the seventh day, MDA began to decrease, and the AChE levels increased (Figure 4a,d, *p* < 0.001), indicating the onset of a recovery mechanism against oxidative damage. Studies have shown that the pathogenesis of As toxicity relates to the dynamic balance of oxidative stress caused by antioxidants and pro-oxidants, and As methylation has long been considered a detoxification process [43]. Excessive reactive oxygen species and oxidative stress induced by As play an important role in As toxicity mechanisms. The levels of antioxidant enzymes such as GST, SOD, and AChE are suitable indirect indicators for the evaluation of the oxidative stress levels of organs.

AChE is a hydrolytic enzyme of acetylcholine that directly participates in the regulation of plant neural functions, such as muscle movement, brain thinking, and memory, etc. Studies have shown that iAs significantly inhibits AChE in the brains of fish, which is similar to the results of the initial stage of this study [44]. Following 14 days of exposure, the activities of AChE, SOD, and GST enzymes were gradually inhibited and then gradually recovered to normal levels. At the same time, the MDA content increased almost fourfold, indicating that the crucian carp was still under As(III) stress at this stage, and severe oxidative damage occurred on the fourteenth day. However, as the exposure time was prolonged, the antioxidant defense system could be activated, and the activities of AChE, SOD, and GST also began to increase again in order to reduce the production of reactive oxygen species and MDA [29]. We found that the MDA content decreased on the 28th day compared to the 14th day (Figure 4d, *p* < 0.001), indicating that crucian carp can resist oxidative stress caused by As(III) under environmental pressure by activating the corresponding defense system. At the same time, we also observed similar results on the fourth day, indicating that the crucian carp’s antioxidant defense system was not all activated at once during the entire stress period, but rather gradually activated.

**Figure 5:**
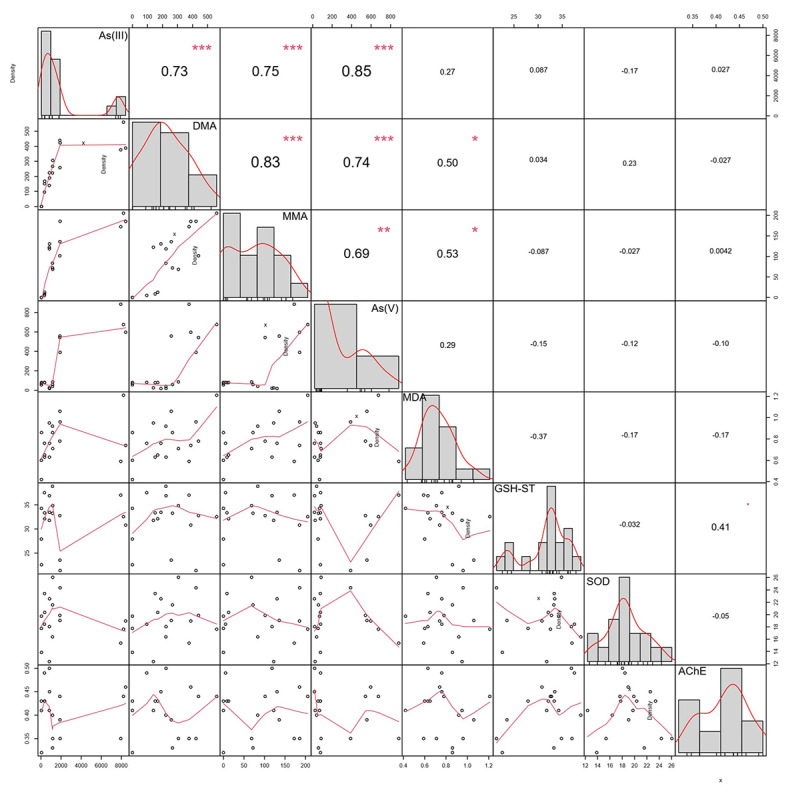
Correlations between enzymatic activity and arsenic species content in the liver of crucian carp chronically exposed to 1 mg/L waterborne As(III) for 28 d. * represents *p* < 0.05, ** represents *p* < 0.01, and *** represents *p* < 0.001.

MDA is a byproduct of lipid peroxidation in living organisms, and is an important indicator for assessing the degree of cell damage [45]. As shown in Figure 5, there is a significant positive correlation among As species in the liver of crucian carp under 1 mg/L As(III) stress (*p* < 0.01), indicating biological transformations between As species that occur in the liver after entering the fish body. This indicates that, after entering the fish body, As(III) undergoes biotransformation in the liver, leading to an increase in MDA content and a series of enzyme-catalyzed reactions (Figure 4). There is a significant positive correlation between AChE and GST enzymatic activities in the liver (*p* < 0.05). Furthermore, this study also found a significant positive correlation between MDA content and organic As compounds (DMA and MMA) due to long-term As(III) exposure (*p* < 0.05), which may imply oxidative damage caused by the methylation process of As in the fish body. A significant correlation has been observed between As accumulation in mirror carp (*C. carpio*) and elevated MDA levels following waterborne As exposure, but no significant association was found between MDA and As species content in a separate experiment focusing on foodborne As exposure [46,47]. The ability of fish to biotransform As and respond to oxidative stress is influenced by the method of intake; foodborne As exposure is generally absorbed and transformed via the intestine, whereas waterborne As exposure also includes skin and gills. Although whether As methylation detoxifies or enhances As toxicity is still a continuous debate, our current study found a significant positive correlation between As methylation and lipid peroxidation damage, which has rarely been reported in other studies [12,31,47].

## 4. Conclusions

The study investigated the sensitivity of four freshwater aquatic organisms to acute iAs exposure, examining the accumulation and transformation patterns of As in various tissues of crucian carp under chronic exposure. The findings revealed that Chinese mitten crab is more sensitive to As(III) than the other three fish species, while loaches showed relative tolerance to As(V). The accumulation and biotransformation process of As species induced typical antioxidant stress defense mechanisms in the liver, resulting in oxidative damage to crucian carp. Overall, crucian carp showed a strong tolerance to As(III), and, during exposure, the body underwent a series of metabolic and enzyme-catalyzed reactions to counteract As accumulation. Significantly, the methylation process of As triggers lipid peroxidation damage, and its toxic mechanism requires further investigation. The present research provides valuable information regarding the toxicological assessment of iAs.

## Figures and Tables

**Table 1:** Acute toxicity exposure mortality with concentration log linear regression equation.

Species Scientific Name	Time	As(III)		As(V)	
		Regression Equation	R^2^	Regression Equation	R^2^
*M. anguillicaudatus*	24 h	y = −4.75 + 2.20x	0.924	y = −8.76 + 3.03x	0.989
	48 h	y = −4.25 + 2.57x	0.984	y = −9.27 + 3.43x	0.953
	96 h	y = −5.55 + 3.95x	0.997	y = −10.61 + 4.25x	0.987
*P. parva*	24 h	y = −3.59 + 1.97x	0.931	y = −5.64 + 2.22x	0.956
	48 h	y = −3.29 + 2.40x	0.912	y = −5.2 + 2.38x	0.981
	96 h	y = −3.23 + 2.63x	0.979	y = −4.26 + 2.26x	0.980
*E. sinensis*	24 h	y = −1.63 + 1.04x	0.930	y = −4.55 + 2.06x	0.948
	48 h	y = −1.32 + 1.76x	0.969	y = −3.61 + 2.06x	0.979
	96 h	y = −0.64 + 1.74x	0.964	y = −3.26 + 2.26x	0.967
*C. auratus*	24 h	y = −2.53 + 1.39x	0.973	y = −5.6 + 2.63x	0.952
	48 h	y = −2.93 + 2.00x	0.995	y = −6.34 + 2.83x	0.993
	96 h	y = −4.17 + 3.39x	0.969	y = −5.79 + 2.30x	0.919

**Table 2:** LC50, SC values, and 95% confidence values for the As (III) and As (V). *n* = 4.

Inorganic Arsenic	Species Scientific Name	24 h			48 h			96 h			SC
		LC_50_	Lower Limit	Upper Limit	LC_50_	Lower Limit	Upper Limit	LC_50_	Lower Limit	Upper Limit	
As(III)	*M. anguillicaudatus*	112.49	95.55	132.44	46.14	38.38	55.49	26.92	23.40	30.97	2.33
	*P. parva*	50.71	42.48	60.53	25.59	21.08	31.08	17.62	14.92	20.81	1.96
	*E. sinensis*	21.13	15.13	29.53	5.58	4.09	7.62	2.55	1.89	3.45	0.12
	*C. auratus*	48.75	37.63	63.17	29.31	22.78	37.71	17.10	14.10	20.75	3.18
As(V)	*M. anguillicaudatus*	690.24	644.56	739.16	484.17	449.35	521.70	323.59	302.73	345.90	71.47
	*P. parva*	294.42	267.27	324.33	151.00	135.94	167.72	86.89	78.87	95.73	11.91
	*E. sinensis*	126.19	114.42	139.18	57.68	51.75	64.29	33.19	30.09	36.61	3.62
	*C. auratus*	281.17	254.56	310.57	169.42	153.22	187.33	131.51	119.37	144.89	18.45

LC50 = Lethal concentration for 50% of animals. All values in mg/L.

**Table 3:** Kinetic parameters of the uptake and removal of arsenic compounds in crucian carp under As (III) stress.

Tissue	Cw (μg/L)	k_1_	k_2_	BCF	C_Amax_ (μg/kg)	B_1/2_ (d)	R^2^
Muscle	1000	0.015335	0.03727	0.41145	411.45	18.59404	0.88712
Gills	1000	0.412295	0.726	0.5679	567.9	0.954545	0.45647
Intestine	1000	0.159382	0.1855	0.8592	859.2	3.735849	0.87028
Skin	1000	0.059684	0.0262	2.278	2278	26.45038	0.88427
Liver	1000	0.232551	0	-	-	-	0.89525

## Data Availability

The datasets used and analysed during the current study are available from the corresponding author upon reasonable request.

## Supplementary Materials

The following supporting information can be downloaded at https://www.mdpi.com/article/10.3390/toxics12030221/s1: Table S1: Operation parameters of HPLC-ICP-MS (Agilent 7500 cx); Table S2: Summary of analyte masses, elements for internal standard method (ISTD), analytical conditions for octopole reaction system (ORS), correlation coefficient of standard curve(R), limits of detection (LOD) and results of quality control for study elements; Table S3: Acute toxicity test of inorganic arsenic solution concentration (mg/L), *n* = 4.
