# Supplementary material for: Toxic Effects of Arsenic on Four Freshwater Aquatic Species and Its Transformation Metabolism in Crucian Carp (Carassius auratus)
Source: Toxics. 2024 Mar 17;12(3):221. doi: 10.3390/toxics12030221 (PMC10975476; doi:10.3390/toxics12030221)
Supplement: Supplementary file 1 [file toxics-12-00221-s001.zip › toxics-2845309-supplementary.pdf]

Table S1. Operation parameters of HPLC-ICP-MS (Agilent 7500 cx).

ICP-MS conditions (optimized daily)		HPLC conditions	
RF power	1500 W	Flow rate	Isocratic, 0.8 $mL{\cdot}min^{-1}$
Reflected power	<15 W	Injection volume	100 μL
Plasma gas flow rate	15 $L{\cdot}min^{-1}$	Analytical column	PRP-X100 (4.1 mm×250 mm×10 μm. PEEK), Hamilton
Nebulizer gas flow rate	0.98 $L{\cdot}min^{-1}$	Mobile phase	50 mM $NH_4(CO_3)_2$; 0.2 mM EDTA, 1% MetOH. pH = 8.5
Auxiliary gas flow rate	0.24 $L{\cdot}min^{-1}$	Total elution time	13 min
Collision gas H_e	4.0 $mL{\cdot}min^{-1}$		
Makeup Gas	0.15 $L{\cdot}min^{-1}$		
Nebulizer Pump	0.10 rps		
Uptake Speed	0.40 rps		
Uptake Time	45 s		
Stabilization Time	30 s		
Acquisition	Spectrum(Multi Tune)		
Peak Pattern	Full Quant(3)		

Table S2. Summary of analyte masses, elements for internal standard method (ISTD), analytical conditions for octopole reaction system (ORS), correlation coefficient of standard curve(R), limits of detection (LODs) and results of quality control for study elements.

Analyte	Isotope	ISTD	ORS	R	LOD1 ($\mu g\cdot kg^{-1}$)	LOD2 ($\mu g\cdot L^{-1}$)	RSD (%)	Nominal values ($mg\cdot kg^{-1}$)	Found value ($mg\cdot kg^{-1}$)	Recovery (%)
As	75	^{72}Ge	He	0.9999	3.0	0.50	8.16	4.8 ± 0.3[a]	4.66 ± 0.21	92.8-101.5
As(III)	/	/	No gas	0.9997	4.5	0.27	5.29	0.05[b]	0.0497 ± 0.0020	95.5-103.6
As(V)	/	/	No gas	0.9998	5.0	0.57	4.69	0.05[b]	0.0455 ± 0.0031	85.2-96.8
DMA	/	/	No gas	0.9985	3.2	0.64	6.68	0.340 ± 0.051[a]	0.27 ± 0.023	72.5-86.3
MMA	/	/	No gas	0.9998	1.5	0.61	3.58	0.05[b]	0.0497 ± 0.0032	93.1-105.8

LOD1 represents the detection limit of arsenic species in crucian carp tissues, while LOD2 represents the detection limit of arsenic species in water.

The data are represented as means ± standard deviation.

[a]: BCR-627 tuna tissue, [b]: Spiked test sample in 0.050 $mg\cdot kg^{-1}$

Table.S3. Acute toxicity test of inorganic arsenic solution concentration (mg·L^{-1}), n = 4.

Group	Elements	Blank Control (μg·L^{-1})	Test 1	Test 2	Test 3	Test 4	Test 5	Test 6	Spiked test sample (μg·L^{-1})
Initial concentration	As(III)	ND	1.02	2.14	4.75	10.41	22.88	50.44	10.0
	As(III)	ND	0.97	2.36	4.93	10.43	22.15	52.02	9.72
	As(III)	ND	1.01	2.11	4.82	10.50	23.49	48.96	9.46
	As(III)	ND	1.00	2.20	4.83	10.45	22.84	50.47	10.5
End (12H)	As(III)	ND	0.96	2.22	4.69	10.32	22.1	50.05	10.0
	As(III)	ND	0.97	2.13	4.85	10.29	21.59	48.71	9.28
	As(III)	ND	0.99	2.1	4.44	10.44	21.85	47.52	9.39
	As(III)	ND	0.97	2.15	4.66	10.35	21.85	48.76	10.3
Initial concentration	As(V)	ND	21.05	32.01	50.99	80.36	130.6	210.54	91.0
	As(V)	ND	20.52	31.05	52.37	78.25	122.3	208.79	98.7
	As(V)	ND	19.88	32.06	49.78	77.31	129.5	203.54	103
	As(V)	ND	20.48	31.71	51.05	78.64	127.47	207.62	104
End (12H)	As(V)	ND	19.88	31.56	51.33	80.1	128.2	199.2	95.5
	As(V)	ND	20.37	30.31	48.25	78.98	120.9	210.6	92.8
	As(V)	ND	20.22	30.98	49.76	79.22	122.3	198.7	103
	As(V)	ND	20.16	30.95	49.78	79.43	123.8	202.8	95.8

ND: not detectable.
